# A Weakly Supervised Semantic Segmentation Model of Maize Seedlings and Weed Images Based on Scrawl Labels

**DOI:** 10.3390/s23249846

**Published:** 2023-12-15

**Authors:** Lulu Zhao, Yanan Zhao, Ting Liu, Hanbing Deng

**Affiliations:** 1 College of Information and Electrical Engineering, Shenyang Agricultural University, Shenyang 110866, China; zhaolulu_zll@163.com (L.Z.); zynzhaoyanan@163.com (Y.Z.); syau_amipu@163.com (T.L.); 2 Liaoning Agricultural Informatization Engineering Technology Research Center, Shenyang 110866, China

**Keywords:** image segmentation, semantic segmentation, weakly supervised learning, scrawl label

## Abstract

The task of semantic segmentation of maize and weed images using fully supervised deep learning models requires a large number of pixel-level mask labels, and the complex morphology of the maize and weeds themselves can further increase the cost of image annotation. To solve this problem, we proposed a Scrawl Label-based Weakly Supervised Semantic Segmentation Network (SL-Net). SL-Net consists of a pseudo label generation module, encoder, and decoder. The pseudo label generation module converts scrawl labels into pseudo labels that replace manual labels that are involved in network training, improving the backbone network for feature extraction based on the DeepLab-V3+ model and using a migration learning strategy to optimize the training process. The results show that the intersection over union of the pseudo labels that are generated by the pseudo label module with the ground truth is 83.32%, and the cosine similarity is 93.55%. In the semantic segmentation testing of SL-Net for image seedling of maize plants and weeds, the mean intersection over union and average precision reached 87.30% and 94.06%, which is higher than the semantic segmentation accuracy of DeepLab-V3+ and PSPNet under weakly and fully supervised learning conditions. We conduct experiments to demonstrate the effectiveness of the proposed method.

## 1. Introduction

Weeds will compete with crops for nutrients such as fertilizers and sunlight, thus affecting the crop growth and yield. The rapid and effective removal of weed hazards is of great significance to improve crop yield and quality. Using deep learning to accurately identify weeds can ensure crop yield and growth security with the goal of pesticide reduction, efficiency increase, and safety. In 2006, Hinton et al. [1] proposed the use of a fully supervised fine-tuning method to solve the problem of the gradient vanishing in deep network training, which provided an efficient solution for the training of multilayer network models and started the wave of deep learning. With the wide application of computer vision technology based on deep learning models in the field of crop phenotyping, key vision technologies such as image classification, object detection, and image segmentation have rapidly promoted the development of related research in the field of crop phenotyping. In particular, the image semantic segmentation technique, thanks to the model’s ability to accurately segment the image at the pixel scale, plays an important role in the measurement of crop canopy cover [2], leaf area index [3], and planted area estimation [4].

Currently, deep learning technology in the field of agriculture is mainly based on fully supervised learning as the main training mode. Using manually labeled learning samples, the deep network model can fully learn the main features in the samples and give the optimal solution for each type of prediction task. The combination of deep learning and UAV remote sensing [5] is also widely used in agriculture. For plant disease recognition through UAV, the artificial neural network (ANN) trained by Ahmadi et al. [6] showed a strong discriminant ability. Researchers also make full use of the advantages of deep learning technology, based on deep convolutional networks, to obtain key features in plant images and complete weed segmentation with high precision. For example, Fathipoor et al. [7] used deep convolutional neural networks to segment crops and weeds in agricultural fields and demonstrated that convolutional neural networks can detect small weeds and that their structures are more suitable for weed recognition early in the growing season. Genze et al. [8] used a residual neural network as a feature extractor to detect and segment weeds in sorghum fields and further published a manually annotated and expert-curated UAV image dataset that was able to exhibit good weed detection performance under motion-blurred capture conditions. Guo et al. [9] used a lightweight network based on an encoder–decoder architecture with randomized split separable residual blocks to compress the model while increasing the number of network layers to extract richer pixel category information, which was optimized by a weighted cross-entropy loss function to improve the segmentation accuracy and real-time segmentation speed of the crop weed dataset. Jiang et al. [10] developed a deep learning-based semantic segmentation model using Visual Transformer to classify and localize weed regions in grassy areas, which is capable of detecting weeds in a recovered grass environment. The effectiveness of deep learning techniques in weed detection can be seen.

However, fully supervised deep learning methods have high requirements for training samples, especially for image segmentation tasks, which require pixel-level mask labels to support the whole training process of the model. While the existing public datasets contain only a small number of plant species, which cannot meet the demand for crop phenotypic diversity, researchers have to build the training set according to their tasks, which requires a large amount of manual labor, especially for a large number of agricultural images, and it is difficult to achieve pixel-level mask labeling for all samples. Therefore, to reduce the dependence on accurate pixel-level labels, some researchers have attempted to use weakly supervised learning and unsupervised learning models for the tasks of detection and segmentation of agricultural plant phenotypes. Andres et al. [11] proposed a real-time crop and weed classification detection method that requires only color images, which runs at a fast rate and is capable of real-time detection tasks. Chen et al. [12] achieved effective segmentation of UAV images of maize Northern Leaf Blight based on image-level labels combined with an auxiliary branching module and a feature multiplexing module. Wang et al. [13] used a LandSat8 remote sensing dataset and single-pixel labels involved in UNet model loss calculation for weakly supervised farmland image segmentation, which reduced the cost of labeling the dataset to a certain extent and maintained an image segmentation accuracy that was close to the real farmland data. Kim et al. [14] proposed a weakly supervised crop area segmentation model to effectively identify uncut crop areas for path guidance. This method uses images of specific areas to train the classification model to segment the target area from the input image based on implicit learning positioning.

For the image segmentation task, the unsupervised learning method does not give any label information during the training process, but performs clustering operations on similar images, or similar pixels in an image, using clustering [15,16]. The clustering process mainly takes into account the color and texture of the image and uses the variance in the pixel values in the neighborhood to determine the degree of similarity between the images or pixels. However, for images dominated by plants, the target objects (plants in the foreground) and nontarget objects (plants in the background) have a high similarity in color and texture, and it is difficult to achieve the distinction between foreground objects and plants in the background by relying on clustering methods only. Compared with unsupervised learning, weakly supervised learning methods can provide richer information about the target object during model training, while the cost of creating labels is much lower than for fully supervised methods [17].

Currently, image segmentation methods based on weakly supervised learning mainly use four types of labels: image labels [18], bounding box labels [19], point labels [20], and scrawl labels [21]. Image-level labeling has the lowest labor cost, but the labeling information is mainly used to distinguish pixel regions with similar colors and textures, which leads to the need for a large number of effective datasets to support the experiment due to the extreme lack of pixel-level supervisory information; bounding-box labeling is characterized by fast labeling, and the labeled region contains the complete target object, and the coordinates of the four vertices of the bounding box are utilized to determine the object labeling location, but this method cannot determine the boundary of the foreground object in detail, and the labeling region contains a lot of background information, affecting the model in terms of the segmentation accuracy near the target contour; point labeling is characterized by the labeling region location being accurate and not containing background information, but compared with the first two forms of labeling, the lack of overall information on the target to describe means that it is easy to lose the features in the process of feature extraction. Scrawl labeling is an annotation method that uses lines to label the target object, which can obtain the positional information and the shape characteristics of the target object and reduce the influence of the noise information in the background on the target area, and its manual labeling cost is approximate to that of bounding box labeling.

The use of different label types in crop phenotyping research varies greatly for semantic segmentation tasks such as distinguishing crop plants and weeds, considering that weeds are characterized by irregular morphology and regional aggregation, while the scrawl approach can separately label maize plants and weed regions with labels of arbitrary shapes and does not bring background information into the training process. Therefore, we propose a Scrawl Label-based Weakly Supervised Semantic Segmentation Network (SL-Net), which utilizes a pseudo label module to generate scrawl labels and adopts a lightweight network used to gradually reduce the number of parameters and speed up feature extraction. The spatial pyramid pooling module is used to extract and fuse the contextual information, the global information is obtained to obtain the deeper features, the loss function is optimized, and the final result is obtained to obtain the semantic segmentation result of the image with high accuracy. We also compare the SL-Net model with the DeepLab-V3+ and PSPNet model in depth in later experiments.

The contributions of our work can be summarized as follows:

- We propose a pseudo label generation module, which generates bipartite maps of maize and weeds by using Exceed green feature Graying (EXG) and Otsu, generates maize masks by the GrabCut method, and obtains the mask images of maize and weeds, respectively, by using the dissimilar-or operation, and then refines the generated pseudo labels by the erosion and DenseCRF methods to reduce the annotation cost.
- We design an encoder–decoder-based semantic segmentation network, introducing the lightweight model MobileNet-V2 and the Atrous Spatial Pyramid Pooling (ASPP) module to obtain high-accuracy semantic segmentation results.
- We demonstrate the feasibility of the proposed method by comparing it with existing methods. The proposed method outperforms the semantic segmentation accuracy of DeepLab-V3+ and PSPNet under weakly and fully supervised learning conditions, and experiments on the corn and weed image dataset demonstrate the effectiveness of our model.

This paper is organized as follows: In Section 1, the significance of semantic segmentation in the field of plant phenotypes is introduced, the problems and challenges of fully supervised semantic segmentation models are explained, and the characteristics of different pseudo labels are introduced. Section 2 introduced a weakly supervised semantic segmentation method based on scrawl labeling, including the pseudo label generation method and the model framework. Section 3 is the experiment and discussion section, which describes the experimental environment, as well as the loss function and evaluation metrics and summarizes and analyzes the training and testing results of our method; in addition, we compare the results of this paper’s model with those of DeepLab-V3+ and PSPNet models in weakly supervised and fully supervised learning scenarios. Section 4 is a summary of this article.

## 2. Materials and Methods

### 2.1. Datasets and Labels

#### 2.1.1. Data Acquisition and Preprocessing

This experiment was carried out with the maize cultivar “Xianyu 335”, which is highly resistant to stem rot and moderately resistant to powdery mildew, Campylobacter leaf spot, maize stem borer, big spot disease, etc. Its diverse disease-resistant qualities can reduce the probability of disease in the seedling stage and maintain the integrity of the plant growth. In the data acquisition stage of the experiment, a conventional optical camera (Canon 6D, Tokyo, Japan) was used to photograph a single maize plant from the top view direction, and the image acquisition was performed at the seedling stage (3~5 leaves) of the maize, with the height of the top view shot at 1~1.2 m; the original separate rate of the image was 5472 × 3648 pixels, and the image size was standardized to 1024 × 1024 pixels after image cropping and scaling.

To ensure the randomness of the weeds in the original images, no weeding was performed in this experiment between the maize emergence stage and the nodulation stage. In addition, to adequately train the deep learning model, the experiment adopts the data enhancement method, in which the original images are expanded with three methods of mirror flipping, rotating left or right by 90°, and pretzel noise, respectively, to the original samples, to enhance the generalization ability of the model after training. The original images in the experiment were 300 and 900 images and were obtained by data enhancement, and all 1200 images were randomly sampled in a ratio of 8:1:1 to form a training set (960 images), a test set (120 images), and a validation set (120 images).

#### 2.1.2. Scrawl Label

**Figure 1:**
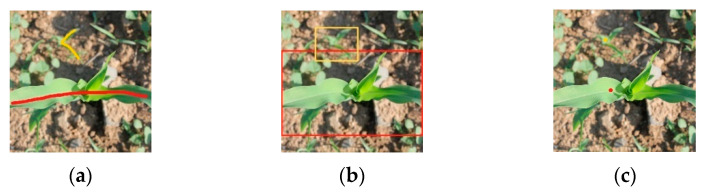
Three weak labels of maize and weed images (Red is used for marking maize, yellow is used for marking weeds): (**a**) Scrawl labels; (**b**) bounding box labels; (**c**) point labels.

Due to the high cost of manual semantic labeling, to reduce the cost of labeling and at the same time ensure that the labels can better describe the morphological characteristics of different categories of plants (maize or weeds) in the image, this study utilizes scrawl labels to complete the work of labeling maize or weed objects in the image. The schematic diagram of scrawl labeling is shown in Figure 1a, where maize plant areas and weed areas are labeled in the image using manually drawn lines, respectively, where red lines indicate that the pixel areas where the lines are located and the related neighborhoods are pixels of maize plants, and yellow lines indicate that the pixel areas where the lines are located and the related neighborhoods are pixels of weeds.

Since the direction in which the lines are drawn is unrestricted, scrawl labels can be changed at will depending on the morphology of the plant itself. Compared with the weak labeling of the bounding box type (Figure 1b), scrawl labels do not contain background information and do not provide the model with wrong pixel classifications during training. For the weak labeling of single-pixel-point types (Figure 1c), although the annotation method is less costly than scrawl labeling, the point label gives too little pixel information to express the texture and color information of the plant surface in terms of the shape of the target object, the growth mode, etc. For the category labeling at the image level, the categorical labeling of the image does not give information about the location of the foreground in the image. It can be seen that scrawl labels can give maximum information about the texture, color, and location of the plant without generating pixel misclassification.

### 2.2. SL-Net Modeling Framework

**Figure 2:**
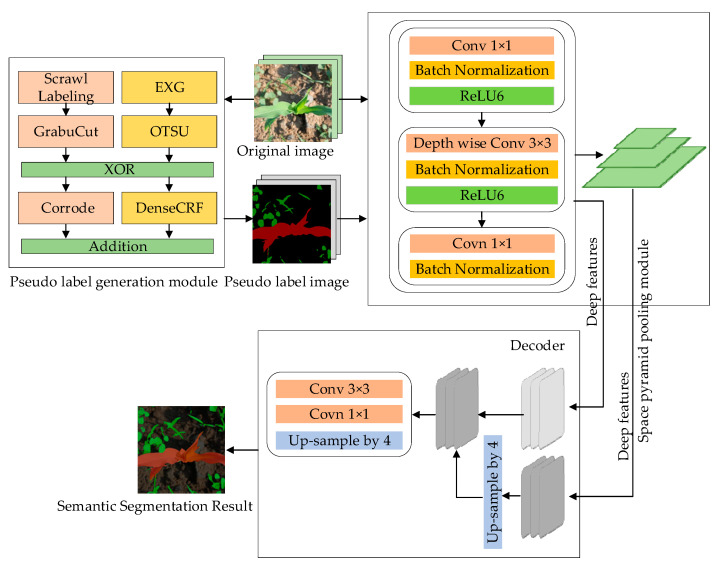
Framework of SL-Net (The red area is maize, and the green area is weeds).

The overall framework of the SL-Net model (shown in Figure 2) is mainly divided into two parts: the first part is based on the pseudo label generation module as the core, where the images with scrawl labels are input to the pseudo label generation module, and the corresponding pseudo labels of the image are obtained by using the methods of threshold segmentation, graph cut, and conditional random field; the second part utilizes an encoder–decoder structure with a deep convolutional network as the core to use the images as well as the corresponding pseudo labels of the images as the training samples, and the pixel information corresponding to the pseudo labels is utilized to optimize the network parameters in the model in training to achieve semantic segmentation of the images of maize and weeds at the end of the training.

To verify the effectiveness of the model framework, this study refers to the encoder–decoder structure of the DeepLab-V3+ [22] model, where the encoder categorizes and analyzes the inputs into feature maps through convolution, pooling, and other operations and obtains the higher-order semantic information; in the decoder, the feature information is transformed into the target semantic information through the operations of up-sampling, convolution, and other operations, and the obtained target information and location information is projected onto the specific pixels to obtain the predicted classification information.

In this study, the encoder and decoder were optimized based on the DeepLab-V3+ model, respectively. Deeply separable convolution was introduced in the encoder to increase the receptive field, gradually reduce the feature map, and obtain more semantic information to strengthen the features, the spatial information was recovered by using the decoder module to improve the segmentation accuracy, and the encoder feature extraction was carried out by using the feature extraction network and the empty spatial pyramid pooling (ASPP) module in MobileNet-V2 to use the feature vectors obtained as shallow features. In the decoding stage, the deep features and shallow features were combined, and the final feature map was restored to the same size as the input image by one convolution operation and one up-sampling operation, and the semantic segmentation results were finally obtained.

#### 2.2.1. Pseudo Label Generation Module

**Figure 3:**
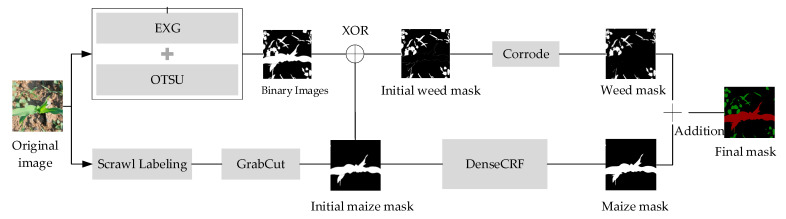
Pseudo label generation process (The red area is maize, and the green area is weeds).

To use scrawl labels more fully in training, this paper adds a pseudo label generation module to the SL-Net model, using pseudo labels generated by scrawl labels to participate in model training instead of manual labels, and the pseudo label generation process is shown in Figure 3.

The maize pseudo labels in this study are optimally obtained based on the Grabcut algorithm and DenseCRF algorithm. The Grabcut [23] algorithm is a foreground extraction algorithm based on minimum user interaction designed by Carsten et al. The algorithm constructs the energy function using both region and boundary terms, and Formula (1) defines the energy function *E*:

(1) $$E\left(\underline{\alpha },k,\underline{\theta },z\right)=U\left(\underline{\alpha },k,\underline{\theta },z\right)+V\left(\underline{\alpha },z\right)$$

where *α* denotes the label of the pixel point covered by the scrawl label, *k* denotes the number of Gaussian components, *θ* denotes the parameter included in the model and the range of values of the parameter, *z* denotes any pixel point in the image, function *U* denotes the regional data term of the energy function as shown in Formula (2), and function *V* denotes the boundary term of the energy function as shown in Formula (3):

(2) $$U\left(\underline{\alpha },k,\underline{\theta },z\right)=\sum_{n}D\left(\alpha_{n},k_{n},\underline{\theta },z_{n}\right)$$

where *n* denotes the pixel serial number in the image, *D* is a hybrid multi-Gaussian model indicating the probability that a pixel belongs to the foreground or background, and the data term function *U* is used to measure the similarity index between the pixel and the foreground or background model so that the region of the foreground or background corresponds as much as possible to the pixel in the original input image, which makes the classification result more accurate.

(3) $$V\left(\underline{\alpha },z\right)=\gamma \sum_{\left(m,n\right)\in C}\left[\alpha_{n}\neq \alpha_{m}\right]\exp^{-\beta {\left\|z_{m}-z_{n}\right\|}^{2}}$$

where *γ* is equal to 50 by default, m and *n* denote two adjacent pixels, *C* is the set of adjacent color pairs, [*α_n_* ≠ *α_m_*] is 1 when pixel m and pixel *n* are on the boundary of the region and 0 otherwise, the parameter *β* is the weight value of the boundary term, which is determined by the contrast of the image, and the *V* function of the boundary term is used as a measure of the similarity of the categories of the neighboring pixels and promotes the smoothing of the target region, making the classification results more continuous.

In image presegmentation using scrawl labels, the default range of the labeled region is the whole image. Firstly, all pixel points *z_n_* in the image are initialized with labels, the labels *α_n_* of the pixel points that are covered by the labeled lines are initialized to 1 and put into the foreground pixel subset, and the labels of the rest of the pixel points are initialized to 0 and put into the background pixel subset. Then, the function *D* is used to calculate the probability that pixel *n* belongs to the foreground and the probability that it belongs to the background and compare the size of the two; if the value of the probability of belonging to the foreground is bigger, it means that pixel *n* belongs to the foreground and can make the value of the energy function E smaller so that it is classified into the foreground pixel subset, and vice versa, it is put into the background subset. Parameters *m* and *n* are neighboring pixels in the *V* function, and the value obtained by calculating the Euclidean distance is used to judge the difference between the two pixels. The closer the value is to 0, the greater the difference between two pixels is. This means that these two pixels with different categories may be at the edge of segmentation. In this experiment, the above process is iterated five times to obtain the initial maize mask image with maize as the foreground region.

Since the segmentation results obtained by Grabcut have low accuracy at the edges of the plant, to improve the labeling accuracy, this study uses the DenseCRF [24] algorithm for optimization. The DenseCRF algorithm consists of a unitary potential function and a binary potential function. The use of a unitary potential function measures the probability that the observed value of pixel *i* is *y_i_,* but its category label is *x_i_*, and the value of pixel *i* is related to the shape of the pixel and its texture, position, and color; the relationship between the nodes *p* is the pixel point position, and the relationship between pixels are closely linked using the binary potential function, which measures the probability that the color value of adjacent pixel points belongs to the same category and solves the problem of a fuzzy boundary of the classification target, and *i* is the RGB value of the pixel point. Its energy function is shown in Formula (4):

(4) $$E\left(x\right)=\sum_{i}\varphi_{u}\left(x_{i}\right)+\sum_{i}\varphi_{p}\left(x_{i},x_{j}\right)$$

where *x_i_* denotes the feature of the *i*th pixel, *x_j_* denotes the feature of the *j*th pixel, usually information such as pixel point location and pixel value; *ϕ_u_* and *ϕ_p_* are two potential functions, and *ϕ_u_* denotes the relationship between individual pixels, while *ϕ_p_* denotes the relationship between neighboring pixels. When the value of the function *E*(*x*) is smaller, the predicted category label *x* is more accurate, and by iteratively minimizing the energy function, the pixels in the initial mask image are more accurately classified into category labels, so that the segmentation edges are smoother and closer to the real boundaries and the optimized maize-labeled images with only maize plants are obtained.

**Figure 4:**
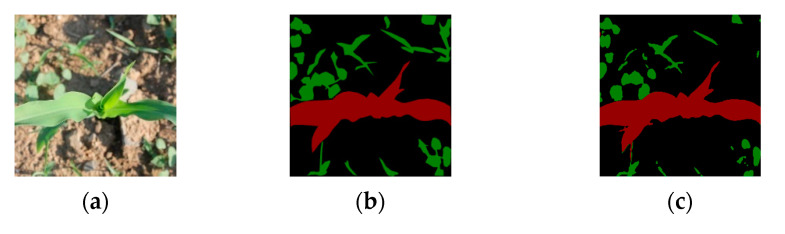
Pseudo label with ground truth (The red area is maize, and the green area is weeds): (**a**) original Image; (**b**) ground truth; (**c**) pseudo label.

The weed label images are then obtained by superimposing the preliminary weed mask through the Exceed green feature Graying (EXG) method with the Otsu method and the difference method and then optimized by the corrosion algorithm. Finally, the maize label images are superimposed with the weed label images to synthesize the pseudo label images. Since the position of weeds in the image is relatively random, but the growth area is more concentrated, to more accurately obtain the pseudo-labeled image of weeds, this study uses the EXG method in conjunction with the Otsu [25] to obtain the binary mask images with the information of green pixels in the image, and because of the high color similarity between maize and weeds, the method can obtain the binary mask image containing the maize and the weeds. The binary mask images containing both maize and weeds are obtained by this method, and then, using the maize pseudo labels obtained after scrawl labeling, the pseudo labels containing only weeds can be obtained by the dissimilarity operation. However, the initial weed mask obtained also contains the outline pixels of the maize plant, so an erosion algorithm is used to optimize the processing of the fine edges, and finally, the pseudo label image of the weed is obtained. Figure 4c shows the pseudo label image containing maize and weeds, and the comparison with the true value (Figure 4b) shows that the maize pseudo label is consistent with the true value in terms of mask morphology; the pseudo labels of weeds are very close in pixel morphology, location, density, etc., when compared with the true-value image, and there are also some missing pixels, but due to the large number of weeds and the similarity in morphology, the pseudo labels that have been generated can already satisfy model training needs.

The pseudo label generation method in this experiment uses the EXG, which has a good effect on the extraction of green plant images. In images, shadows, dead grass, and soil can be significantly suppressed, and plants are more prominent, which has a positive effect on the identification of weeds. Due to this characteristic, the quality of pseudo labels may decrease when there are large areas of nongreen plants in the image.

To verify the quality of the pseudo labels, in this paper, cosine similarity [26] and mean intersection over union (MIoU) are used to calculate the similarity between the pseudo label image and the true-value label image. The cosine similarity method represents the image as a vector and measures the similarity of the two images by the cosine value of the angle between the two vectors—the closer the angle is to 0, the higher the similarity is between the two images—as a way of proving the usability of the pseudo-labeled dataset. The calculation formula is shown in Formula (5):

(5) $$\cos\left(\theta \right)=\frac{\sum_{i=1}^{n}A_{i}\times B_{i}}{\sqrt{\sum_{i=1}^{n}A_{i}^{2}}\times \sqrt{\sum_{i=1}^{n}B_{i}^{2}}}\times 100\%$$

where ***A*** and ***B*** denote the attribute vectors of the two images, and ***A****_i_* and ***B****_i_* denote the components in vectors ***A*** and ***B***, respectively. The cosine similarity is in the range of [−1, 1]; if the two vectors point in the same direction, then the cosine similarity is 1; if the two vectors point in opposite directions, then it is −1; and if the two vectors are perpendicular to each other in space, then it is 0, which means that they are independent of each other.

The mean intersection over union (MIoU) can indicate the degree of pixel overlap between two image masks, which is calculated as shown in Formula (6):

(6) $$MIoU=\frac{1}{k+1}\sum_{i=0}^{k}\frac{p_{ii}}{\sum_{j=0}^{k}p_{ij}+\sum_{j=0}^{k}p_{ji}-p_{ii}}\times 100\%$$

where *k* denotes the number of categories, *k* + 1 represents the number of categories to be partitioned with the addition of the background class, *p_ij_* is the prediction of category *i* to category *j*, *p_ji_* is the prediction of category j to category *I*, and *p_ii_* is the prediction of category *i* to category *i*.

In the experiment, the cosine similarity method is used to calculate the similarity value between the pseudo label image and the true-value image, and a similarity value of 93.55% can be obtained; that is, the angle of the vector pinch between the pseudo label and the true value is close to 0, which indicates that the pseudo label image and the true-value image are very similar in terms of the attributes of image pixel location, pixel value, and so on. When calculating the similarity between the pseudo label image and the true-value image using the MIoU method, the MIoU is calculated every ten images, which can show that the MIoU ratio between the pseudo label image and the true-value image is 83.32%, i.e., the masks of the maize plant image and the weed image have a high degree of overlap. The results of the two methods indicate that the similarity between the pseudo-labeled dataset and the true-value dataset is very high, and the pseudo label can be used instead of the true-value label for model training.

#### 2.2.2. Encoder and Decoder Structure

**Figure 5:**
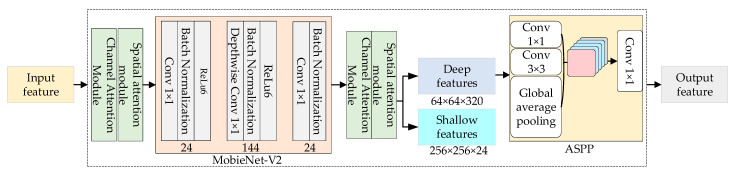
Encoder structure.

In the DeepLab-V3+ model, Xception is the backbone network used for feature extraction, but Xception still cannot meet the requirements of fast segmentation in terms of the total number of parameters and running speed. MobileNet-V2 [27], which is also a lightweight network within the Xception network, has the advantages of a smaller number of parameters and faster operation speed. In this study, MobileNet-V2 replaces the backbone network in the original DeepLab-V3+ model for feature extraction and extracts high-dimensional features through a linear inverse residual structure to obtain high semantic information. As shown in Figure 5, the MobileNet-V2 network in the encoder consists of three parts: the Expansion layer, the Depthwise separable convolution layer, and the Projection layer. Among them, the Expansion layer consists of 1 × 1 convolution, a Batch Normalization Layer, and the ReLU6 activation function, which maps the low-dimensional space to the high-dimensional space through 1 × 1 convolution, normalizes the training data distribution by using the BN layer, reduces the time of adapting to the distribution in each iteration of the network, improves the training speed of the network, and then improves the discriminative accuracy by using the ReLU6 to guarantee the robustness of the network; the Depthwise separable convolution layer is composed of a 3 × 3 Depthwise separable convolution layer, which is composed of a 3 × 3 Depthwise separable convolution layer and a projection layer. The layer is composed of 3 × 3 depth-separable convolution, a BN layer, and an ReLU6 to obtain more features and maintain the data distribution; the projection layer is composed of a 1 × 1 convolution and BN layer, which uses a linear transformation to project the high-dimensional space to the low-dimensional space without using ReLU to filter the information and prevent the nonlinear activation function from losing or destroying the information.

When MobileNet-V2 network undergoes the second subsampling, the feature map input to the backbone network is expanded from 24 dimensions to 144 dimensions through the expansion layer, a depth-separable convolutional layer is used to perform convolutional operations on the 144 channels to extract the features and increase the number of features, and finally, after the dimensionality reduction in the projection layer, the number of feature channels is reduced to 24 dimensions to ensure that the input dimensions are consistent with the output dimensions, so as to ensure that network MobileNet-V2 is under-sampled four times during the training period, and two types of output feature maps can be obtained: one is the feature map with significant semantic information after four subsamplings. The vector dimension of the feature is [64, 64, 320], and the size of the feature map is 1/16 of the original, which is used as the output feature layer of the encoder in SL-Net; and the other is the feature layer with little semantic information, which is used as the output feature layer of the encoder in SL-Net. This layer’s vector dimension after two subsamplings is [256, 256, 24], and the feature map size is 1/4 size of the original map, which will be used as the shallow feature map for the input of the decoder part of SL-Net.

The Atrous Spatial Pyramid Pooling (ASPP) module is in the next layer of the encoder backbone extraction network shown in Figure 5 and consists of three parts: (1) the convolution layer consists of a 1 × 1 convolution and three 3 × 3 dilated convolution, and the three empty space convolutions with parallel expansion rates of 6, 12, and 18, respectively, are used to perform convolution operations on the 256 channels, to expand the receptive field to obtain the corresponding global information and the four convolution operations. A total of four feature maps are obtained, and each convolution operation is followed by a BN layer for normalization; (2) a global average pooling is performed on the features to obtain image-level features, which are convolved point-by-point and then recovered to the original size using bilinear interpolation; and (3) the four feature map channels obtained above are connected and fused by 1 × 1 convolution to obtain a new feature map with 256 channels. The ASPP module convolves the high semantic feature map output from the MobileNet-V2 network with different expansion rates to obtain multiscale features and obtains the deep feature maps with significant semantic information as the output feature maps of the encoder part of the model in this paper, which reduces the time needed by model to learn the data and improves the segmentation accuracy by utilizing the BN layer after each convolutional layer.

**Figure 6:**
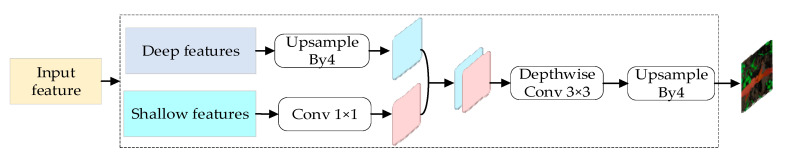
Decoder structure (The red area is maize, and the green area is weeds).

The role of the decoder of the model is to map the acquired features into a high-dimensional space for the classification of each pixel point, and its structure is shown in Figure 6. The decoder has two types of input feature maps: one is the shallow features obtained after two subsamplings, which is reduced from 320 to 48 channels by a 1 × 1 convolution operation, and which has a size that is 1/4 of the original image, because there are too many channels for shallow features, which will lead to a bias towards the shallow features during the training of the network and make the network difficult to be trained; and the other one is the output of the encoder’s deep features, which has a size that is 1/16 of the original image, which is reduced to 4 times and up-sampled bilinearly to the size of 1/1 of the original image, and the size is 1/1 of the original image. The other is the deep feature output from the encoder, the size of which is 1/16 of the original image; the deep feature is bilinearly up-sampled 4 times to 1/4 of the original image, and it is unified with the shallow feature so that the two feature maps with the same resolution are connected, and the feature map after connection is convolved with the 3 × 3 nulls to reduce the loss of spatial features and refine the features without decreasing the sensory field. The feature map is finally restored to the original image size by using the bilinearly 4-fold up-sampling.

## 3. Experiments and Discussions

### 3.1. Experimental Environment and Parameters

The experiment uses Dell Precision-7920 (Round Rock, TX, USA) as the hardware platform for training the model. The platform carries a central processing main frequency of 3.7 GHz, the number of cores is 20, the memory is 64 GB, the model of the computing card is NVIDIA Quadro RTX 5000 (America), and the image processing video memory is 16 GB. The above hardware conditions can satisfy the needs of the model training in this experiment.

The migration learning method [28] is used in training. Firstly, the network is frozen to train using pretraining weights using the generality of multiple classification features, fine-tuning the parameters, and the feature network does not change; then, the network is unfrozen, the whole model is trained, and the feature network is changed to minimize the loss convergence. The specific training parameters are shown in Table 1. The model uses the Adam optimizer and adopts the cosine annealing learning rate decay method to obtain the learning rate, and the upper limit of the whole training cycle is 80. Due to the increase in computational volume after unfreezing the network, to reduce the computational pressure, the number of image batches was set to two images/time.

### 3.2. Loss Functions and Evaluation Indicators

The combined use of the balanced cross-entropy loss function (*L_CE_*) and Dice loss function (*L_Dic_*) allows the net training process to focus on the learning of foreground objects [29]. Therefore, this study uses a pixel-level cross-entropy loss function (*L_CE_*) and Dice loss function (*L_Dic_*) to combine two loss functions. *L_CE_* is used by semantic segmentation in classifying pixel points using softmax, while *L_Dic_* is used as an evaluation metric for semantic segmentation, and the Dice coefficient, an ensemble similarity measure function, calculates the similarity between the predicted results and the true-value samples. The higher the overlap between the predicted and real results, the larger the value. As shown in Formula (7),

(7) $$L=L_{CE}+L_{Dic}=-\frac{1}{N}\sum_{i}\sum_{j=1}^{k}y_{j}^{i}\log\left(p_{j}^{i}\right)+1-\frac{2\left|X\cap Y\right|}{\left|X\right|+\left|Y\right|}$$

where the Dice coefficient takes values in the range of [0, 1], *k* is the number of categories (without background), and the range of *y^i^_j_* is {0, 1}. If the true category of sample *i* is *j*, then *y^i^_j_* =1, otherwise *y^i^_j_* = 0. *p^i^_j_* is the predicted probability that the validation set *i* belongs to the category *j*. *X* is the predicted value of the segmented image, and *Y* is the true value of the segmented image. A larger Dice coefficient and a smaller *L_Dic_* indicate that the datasets are more similar.

In this study, the MIoU (Formula (6)) and the Mean Pixel Accuracy (MPA) of categories are used as evaluation metrics to verify the accuracy of the semantic segmentation, where MPA is calculated as the ratio of the number of correctly categorized pixels for each category, which is calculated as in Formula (8):

(8) $$MPA=\frac{1}{k+1}\sum_{i=0}^{k}\left(p_{ii}/\sum_{j=0}^{k}p_{ij}\right)$$

*k* in Formula (8) is the total number of categories of the objects to be segmented in the foreground, *p_ij_* is the prediction of category *i* as category *j*, and *p_ii_* is the prediction of category *i* as category *i*.

**Figure 7:**
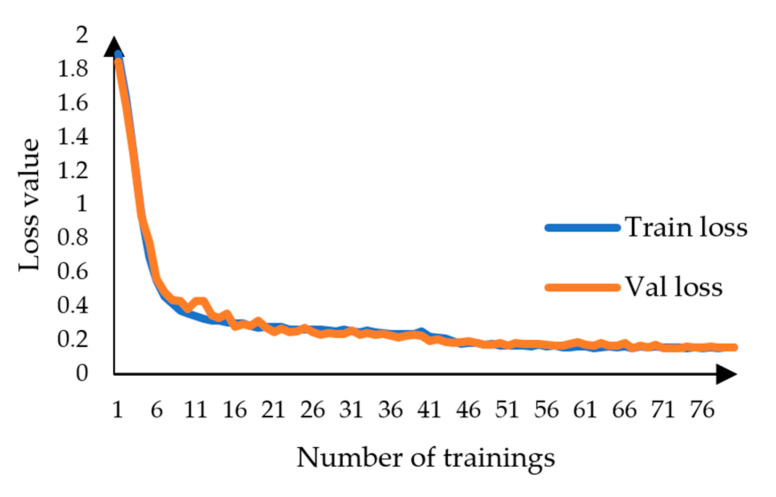
Changes in the loss values of SL-Net.

As shown in Figure 7, the training set and validation set loss curves generated by the SL-Net model after training using the cross-entropy loss function and the cross-entropy loss function based on Dice’s loss, respectively, are plotted. As shown in Figure 5, the loss values converge rapidly and then continue to decline slowly when the network is frozen, and after the network is unfrozen, the losses of the training set and the validation set continue to converge slowly at the same time; the loss function used by SL-Net converges to about 0.25, and there is no phenomenon of sudden increase in loss and overfitting during the training period, which proves that the model network structure is valid.

### 3.3. Experimental Results and Analysis

To verify the effectiveness of SL-Net, DeepLab-V3+ and PSPNet [30] are selected as the comparison models in this experiment, and the ablation test is used to compare them in several aspects, such as the MIoU, the category MPA, and the detection speed fps. Meanwhile, the same improvement is made to the PSPNet model, and the improved model is **PSPNet, which is structured by using the PSPNet model as the base model, the MobileNet-V2 network for feature extraction, and a combined loss function of *L_CE_* and *L_Dic_* for calculating the convergence value. Table 2 shows the experimental results of each model using a pseudo-labeled training set.

Through the experiment, for the change of feature layer, the number of shallow feature map channels that can be obtained from the image after the Xception backbone network is 256, and the number of deep feature map channels that can be obtained is 2048 dimensions. The high dimensionality of the generated feature maps indicates that the size of the parameters in the backbone network is large, which will prolong the training time of the model and increase the training cost. The shallow feature map of the MobileNet-V2 network can be reduced to 24 dimensions, and the deep features can be reduced to 320 dimensions, which shows that the size of the network parameters of MobileNet-V2 is about one order of magnitude smaller than that of Xception; therefore, the amount of computation in the training process is reduced accordingly.

Under the weakly supervised learning condition, the experiment compares the test results of seven models, SL-Net, DeepLab-V3+, *DeepLab-V3+, **DeepLab-V3+, PSPNet, *PSPNet, and **PSPNet, specifically the average intersection and merger ratios, the average pixel accuracies, and the detection speeds, and the data are from Table 2.

In terms of segmentation accuracy, the SL-Net decoder with high semantic features and low semantic features obtains more feature information, which makes the prediction results more accurate, and the model outputs MIoU and mPA reach 87.30% and 94.06%, respectively. Compared with the DeepLab-V3+ series model, the MIoU and mPA of the model are 0.11% and 0.58% higher than those of the optimal model **DeepLab-V3+ in the series, and compared with the PSPNet series model, the MIoU and mPA of the model are 8.25% and 6.9% higher than those of the optimal model *PSPNet in the series, respectively.

**Figure 8:**
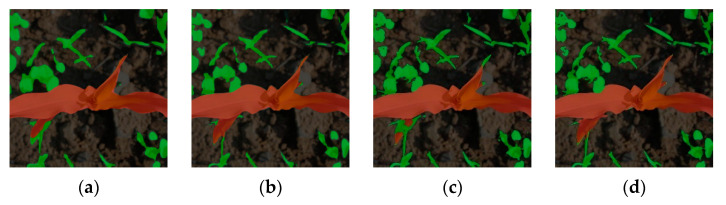
Comparison of prediction results based on weakly supervised training (The red area is maize, and the green area is weeds): (**a**) PSPNet; (**b**) *PSPNet; (**c**) DeepLab-V3+; (**d**) SL-Net.

In terms of model detection speed, SL-Net can reach 2.82 times of the Deeplab-V3+ model (21.2 frames × s^−1^/7.52 frames × s^−1^), which is similar to that of **DeepLab-V3+ (20.32 frames/s); compared with the PSPNet family, SL-Net’s detection speed is faster than that of PSPNet and * PSPNet, but slower than that of **PSPNet, mainly because **PSPNet uses a lower number of parameters, which also leads to a lower segmentation accuracy of **PSPNet than the other networks. The prediction results of different models under weakly supervised learning conditions are shown in Figure 8. Comparing several prediction results of the experiment, SL-Net can segment maize and weeds more accurately.

To verify the effectiveness of the weakly supervised model in the actual segmentation process, this study also compares the segmentation results of the weakly supervised model SL-Net (semantic segmentation accuracy of 87.30% (data from Table 2)) with the semantic segmentation results of the fully supervised model. Table 3 gives the semantic segmentation results of SL-Net with the other fully supervised models, and compared with the fully supervised DeepLab-V3+ series of models, the semantic segmentation accuracy of SL-Net can reach 99.76% for the best model in the series **DeepLab-V3+ and reaches 101.39% of the DeepLab-V3+ model, which indicates that SL-Net and the DeepLab-V3+ series of models for fully supervised learning achieve a similar performance in the semantic segmentation task. Compared with the fully supervised learning PSPNet series model, the semantic segmentation accuracy of SL-Net can reach 103.76% of the optimal model *PSPNet in the series, and compared with the **PSPNet model that is improved in the same way, the ratio of semantic segmentation accuracy reaches 109.02%, which indicates that SL-Net has a better performance on semantic segmentation tasks compared with the fully supervised learning PSPNet model, which has higher accuracy on the semantic segmentation task. In summary, SL-Net exhibits comparable or even better accuracy than the fully supervised model in the semantic segmentation task and achieves a significant performance improvement compared to both the DeepLab-V3+ and PSPNet family of models.

**Figure 9:**
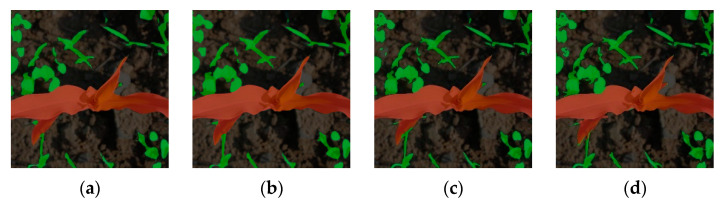
SL-Net compared with prediction results based on fully supervised training (The red area is maize, and the green area is weeds): (**a**) PSPNet (fully supervised); (**b**) *PSPNet (fully supervised); (**c**) DeepLab-V3 (fully supervised); (**d**) SL-Net (weakly supervised).

Figure 9 shows the comparison of the prediction results of the weakly supervised model trained by SL-Net with pseudo labeling and other fully supervised models trained with true-value annotations, from which it can be seen that the DeepLab-V3+ model segmentation results are overall better than the results of the PSPNet model, and no matter whether it is pseudo-labeled dataset training or true-value dataset training, the segmentation results of the model of this paper image with smooth and complete edges of the leaves, fuller segmentation of the maize plant, and close to the labeling of weed segmentation, and the segmentation effect is closer to the true value, which is superior to the segmentation results of the other models.

**Figure 10:**
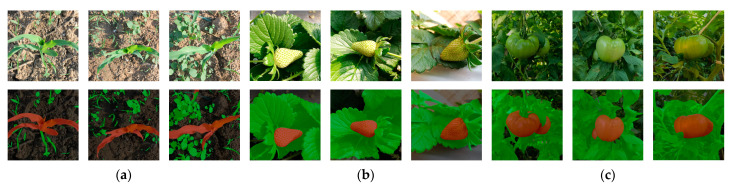
Visualization of SL-Net model segmentation results: (**a**) maize and weed images; (**b**) immature strawberry and leaf images; (**c**) immature tomato and leaf images.

After the experimental comparison, the SL-Net model demonstrated excellent weed segmentation ability and also showed a good performance on other datasets. Figure 10 shows the visualization of the segmentation results of the SL-Net model on a dataset with green similarity, where the first row is the original image, and the second row is the segmented image. According to the visualization results, when the number of weeds in the image changes from low to high ((a): from left to right, columns 1–3), the SL-Net model has a good segmentation performance, and when segmenting immature green fruit and leaf images ((b) and (c): from left to right, columns 4–6 and 7–9), the SL-Net model also has agood segmentation ability.

## 4. Conclusions

In this study, we propose a weakly supervised semantic segmentation model SL-Net based on scrawl labels, which uses pseudo labels instead of manual labels for training. At the same time, the model is lightened to achieve accurate segmentation of seedling maize and weed images in a large field environment. Through the analysis of the experimental process and results, the following conclusions can be obtained:

(1) We designed a pseudo label generation module, which can alleviate the labeling cost of data and improve training efficiency. Using scrawl labels as annotations, pixel-level pseudo labels are generated by combining Exceed green feature Graying (EXG) with DenseCRF conditional random fields, and the MIoU and merger ratio and cosine similarity between pseudo labels and the true values reach 83.32% and 93.55%, respectively.
(2) A weakly supervised semantic segmentation model based on scrawl labels, with pseudo labels instead of manually labeled pixel-level labels as input for model training, using the lightweight network MobileNet-V2 as the backbone network and introducing the ensemble similarity measure function Dice loss function, was developed to achieve high-precision semantic segmentation of seedling maize and weed images. The MIoU and MPA of the model reached 87.30% and 94.06%, respectively, and in the weakly supervised learning mode with pseudo labels as training samples, the MIoU improved by 3.48% and 9.39% compared with the original DeepLab-V3+ model and the PSPNet model, and by 13.32% when comparing with the **PSPNet model, which was improved by the same method.
(3) In the semantic segmentation task of seedling maize and weed images, the SL-Net model can achieve comparable or even better accuracy than the fully supervised model. Compared with the DeepLab-V3+ series of models for fully supervised learning, the semantic segmentation accuracy of the SL-Net model can reach 101.39% of the DeepLab-V3+ model in the series, and compared with the fully supervised learning of the PSPNet series model, the SL-Net model has a higher accuracy in semantic segmentation tasks, in which the highest accuracy of 109.02% is achieved compared to the **PSPNet model in the series.

In summary, the SL-Net model combined with a pseudo label generation module based on scrawl annotation, a lightweight network, and a similarity metric function to realize the semantic segmentation task for seedling maize and weed images in a field environment achieved satisfactory experimental results. This study significantly reduces the time cost of manual annotation, while improving the accuracy and efficiency of agricultural image analysis and providing theoretical and methodological support for agricultural weed segmentation and achieving high-precision, low-cost phenotype analysis of maize seedlings.

## Figures and Tables

**Table 1:** Parameters and initial values of model training.

Parameter Name	Parameter Value
Image batch	2
Max learning rate	0.0005
Min learning rate	0.000005
Training epoch	40
Weight decay	0
Momentum factor	0.9
Input size	1024 × 1024

**Table 2:** Comparison of indicators based on weakly supervised learning.

Model Name	Network of Features	Loss Function	MIoU/%	MPA/%	FPS/Frames × s^−1^
DeepLab-V3+	Xception	*L_CE_*	83.82	90.92	7.52
*DeepLab-V3+	Xception	*L_CE_ + L_Dic_*	84.36	92.04	7.46
**DeepLab-V3+	MobileNet-V2	*L_CE_*	87.19	93.48	20.32
PSPNet	Resnet50	*L_CE_*	77.91	84.41	12.16
*PSPNet	Resnet50	*L_CE_ + L_Dic_*	79.05	87.16	12.17
**PSPNet	MobileNet-V2	*L_CE_ + L_Dic_*	73.98	85.30	38.09
SL-Net	MobileNet-V2	*L_CE_ + L_Dic_*	87.30	94.06	21.20

Note: *DeepLab-V3+ is based on the original DeepLab-V3+ model, adding the *L_Dic_* loss function; **DeepLab-V3+ replaces the Xception backbone network with MobileNet-V2 based on the original DeepLab-V3+ model. *PSPNet is based on the original PSPNet, adding *L_Dic_* loss function to the loss function; **PSPNet is based on the *PSPNet, ResNet50 backbone network replaced with MobileNet-V2.

**Table 3:** Comparison of semantic segmentation accuracy between SL-Net and fully supervised models.

Fully Supervised Model Name	Semantic Segmentation Accuracy/%	Semantic Segmentation Accuracy of SL-Net/%	Accuracy Ratio (SL-Net/Fully Supervised)/%
DeepLab-V3+	86.10	87.30	101.39
*DeepLab-V3+	86.54		100.88
**DeepLab-V3+	87.51		99.76
PSPNet	83.52		104.53
*PSPNet	84.14		103.76
**PSPNet	80.08		109.02

Note: *DeepLab-V3+ is based on the original DeepLab-V3+ model, adding the LDic loss function; **DeepLab-V3+ replaces the Xception backbone network with MobileNet-V2 based on the original DeepLab-V3+ model. *PSPNet is based on the original PSPNet, adding LDic loss function to the loss function; **PSPNet is based on the *PSPNet, ResNet50 backbone network replaced with Mo-bileNet-V2.

## Data Availability

The data presented in this study are available on request from the corresponding author.
